# Long-term outcomes after acute primary angle closure in a White Caucasian population

**DOI:** 10.1186/s12886-015-0100-5

**Published:** 2015-08-19

**Authors:** Walter Andreatta, Ibrahim Elaroud, Peter Nightingale, Maged Nessim

**Affiliations:** Birmingham & Midland Eye Centre, Glaucoma Services, Birmingham, UK; University Hospitals Birmingham NHS Foundation Trust, Birmingham, UK; Wellcome Trust Clinical Research Facility, Queen Elizabeth Hospital, Birmingham, UK; Honorary Senior Clinical Lecturer, University of Birmingham, Birmingham, UK; Queen Elizabeth Hospital, Birmingham, B15 2TH UK

## Abstract

**Introduction:**

Very limited data is available on the morbidity and progression to primary angle closure glaucoma (PACG) in White Caucasian individuals following acute primary angle closure (APAC).

Our aim is to identify the number of eyes who developed PACG following an APAC attack and to determine the risk factors for PACG development in a White Caucasian population in the United Kingdom (UK). We assessed the rate of blindness and visual impairment in the affected eye as defined by the World Health Organisation.

**Methods:**

Retrospective observational study including 48 consecutive eyes of 46 White Caucasian subjects who presented with APAC to a tertiary referral unit in the United Kingdom.

Eyes affected by glaucomatous optic neuropathy at presentation were excluded. We included in our analysis socio-demographic variables, ophthalmic findings, investigations and treatment.

**Results:**

The mean final follow up period was 27 months ± 14 standard deviation (SD). Seven (15 %) eyes developed PACG. Statistical analysis showed that the following factors were linked to a higher risk of progression: length of symptoms before presentation and time taken to break the attack. The intraocular pressure (IOP) was significantly higher in the group who developed PACG at the one- and six-month visit compared to the group which did not develop the disease.

At the final visit 3 (6 %) eyes were blind while 5 (10 %) were visually impaired. PACG was responsible for visual impairment in 2 (4 %) eyes but not for any case of blindness.

**Conclusions:**

Delayed presentation, length of time taken to break the attack and poor IOP control can result in PACG development and visual impairment. APAC causes a low long-term visual morbidity in White Caucasians.

## Backgroud

Primary angle closure glaucoma (PACG) will cause bilateral blindness in approximately 5.3 million people worldwide by 2020 [1]. Acute primary angle-closure (APAC) is a highly symptomatic disease which frequently involves eyes with no previous glaucoma and increases the risk of PACG development.

Extensive research has been conducted over the last two decades on the long-term visual morbidity and progression to PACG in South-East Asian individuals following APAC. However, very limited data are available in White Caucasians.

The aim of our study is to determine the number of eyes that developed PACG following one episode of APAC in a White Caucasian population in the United Kingdom (UK) and the risk factors associated with this blinding disease. In addition, we assessed the long-term visual disability of this cohort as defined by the World Health Organisation (WHO). To the best of our knowledge, this is the first study of this kind in a White Caucasian population since YAG laser peripheral iridotomy became a routine procedure in the management of APAC.

## Methods

This is a retrospective study including 48 eyes of 46 consecutive White Caucasian subjects who presented to the Birmingham & Midland Eye Centre (BMEC) with APAC over a period of two years. No written consent was required. The BMEC is a large supra-regional eye hospital in the United Kingdom offering a 24-hour ophthalmology service. Individuals were identified from the casualty and inpatients databases.

APAC was defined based on the presence of the following consolidated criteria: [2, 3]

1. At least two of these symptoms: ocular or periocular pain, nausea and/or vomiting, headache, a previous history of intermittent blurring of vision with haloes.
2. At least three of the following signs: conjunctival injection, corneal epithelial oedema, mid-dilated unreactive pupil, shallow anterior chamber.
3. An initial intraocular pressure (IOP) higher than 21 mm Hg when measured with a Goldmann applanation tonometer and angle closure on gonioscopy (defined as iridotrabecular contact in three or more quadrants).

Patients presenting with secondary angle-closure, established glaucoma and from ethnic origins other than White Caucasian were excluded from the study.

All APAC patients were initially treated according to our hospital’s protocol which advocates medical management to rapidly reduce the IOP followed by bilateral YAG laser peripheral iridotomies. Further interventions performed in our series in cases with persistently raised IOP included argon laser peripheral iridoplasty (ALPI), diode laser cycloablation, trabeculectomy and lens extraction with or without goniosynechialysis.

We collected socio-demographic data, ophthalmological findings, investigations and interventions performed during the study period. Best-corrected visual acuity (BCVA), IOP and disc appearance were recorded at presentation, one week, one months, six months, one year after presentation and at the last documented visit. Visual fields (VF) were assessed using static automated threshold perimetry (Humphrey Instruments, program 24–2 SITA Fast, Dublin, CA). All patients had at least one reliable VF test with fixation losses less than 20 % and false positive and negative responses less than 33 %. VF defects were confirmed on at least two separate HVF tests. Glaucomatous VF defects were graded as early, moderate and severe according to the Hodapp-Anderson-Parrish criteria [4].

The primary outcome was to identify the number of eyes who developed PACG following an APAC attack and to determine the risk factors leading to glaucoma in White Caucasians. Our secondary outcome was to determine the rate of blindness (BCVA <3/60 and/or a VF < 5 degrees from the point of fixation) and visual impairment (BCVA <6/18 but ≥3/60 and/or a VF < 10 degrees from the point of fixation) in the affected eye as defined by the 2010 version of the World Health Organization International Statistical Classification of Diseases and Related Health Problems [5].

PACG was defined by the presence of glaucomatous optic neuropathy and VF loss as well as iridotrabecular contact in three or more quadrants on gonioscopy [2]. Primary angle closure (PAC) eyes have the same gonioscopy findings as in PACG but no sign of glaucoma [2].

Statistical analysis was completed using PASW Statistics 18 (SPSS Inc., Chicago, Illinois). The statistical test adopted is highlighted next to the p-value in the Results section. A p-value less than 0.05 was considered as statistically significant.

This study adhered to the tenets of the Declaration of Helsinki and was reviewed by the National Research Ethics Service Committee (UK) who ruled that approval was not required.

## Results

Forty eight eyes of 46 subjects were included in the study. 35 (76 %) subjects were female and 11 (24 %) male. The mean age was 69 years ± 12 standard deviation (SD). Two subjects presented with bilateral APAC. The mean final follow up period was 27 months (±14 SD).

Table 1 reports the BCVA documented at the various visits.

**Table 1:** Snellen best-corrected visual acuity of APAC eyes at the clinic visits (n:48)

Snellen BCVA	Presentation	One week	One month	Six months	One year	Final (27 months ± 14 SD)
≥6/6	1 (2 %)	6 (13 %)	11 (23 %)	6 (13 %)	9 (19 %)	10 (21 %)
6/9 – 6/12	8 (17 %)	25 (52 %)	23 (48 %)	35 (73 %)	33 (69 %)	31 (65 %)
6/18 – 6/24	11 (23 %)	5 (10 %)	6 (12 %)	4 (8 %)	3 (6 %)	2 (4 %)
6/36 – 6/60	9 (19 %)	8 (17 %)	5 (10 %)	1 (2 %)	1 (2 %)	2 (4 %)
<6/60	19 (39 %)	4 (8 %)	3 (6 %)	2 (4 %)	2 (4 %)	3 (6 %)
<6/18 and ≥ 3/60	19 (39 %)	11 (23 %)	7 (15 %)	4 (8 %)	3 (6 %)	4 (8 %)
<3/60	10 (21 %)	4 (8 %)	3 (6 %)	2 (4 %)	2 (4 %)	2 (4 %)

*APAC* acute primary angle closure, *BCVA* best-corrected visual acuity

During the study period, 7 (15 %) eyes developed PACG. The mean MD of the final HVF was 8.2 dB ± 2.8 SD. Two eyes developed early VF defect, three moderate and two severe by the last visit.

At the final visit 5 (10 %) eyes had visual impairment while 3 (6 %) were blind. PACG was responsible for visual impairment due to severe visual field constriction in 2 (4 %) eyes but was not accountable for any case of blindness. Other causes of visual impairment were age-related macular degeneration (AMD), epiretinal membranes (ERM) and amblyopia. Blindness was due to AMD in two eyes while in one eye it was secondary to corneal decompensation.

Tables 2 and 3 highlight the IOP and disc appearance in the APAC eyes at the various visits. There was significant difference in IOP at the one- and six-month appointment between the eyes who developed PACG and those who did not (Table 2). Subsequently, the cup-disc ratio (CDR) increased significantly in the PACG group at the six-month and one year appointment (Table 3).

**Table 2:** Mean IOP (±SD) in mmHg measured at the clinic visits

Time from APAC	Non PACG (n:41)	PACG (n:7)	P-value (t test)
Presentation	52 (±10)	56 (±12)	0.421
One week	15 (±9)	14 (±4)	0.689
One month	15 (±9)	21 (±5)	0.009*
Six months	15 (±3)	19 (±5)	0.006*
One year	14 (±2)	14 (±4)	0.652
Final appointment (27 months ± 14 SD)	14 (±2)	14 (±2)	0.887

*IOP* intraocular pressure, *SD* standard deviation, *APAC* acute primary angle closure, *PACG* primary angle closure glaucoma

* Indicates statistical significance

**Table 3:** Mean CDR (±SD) measured at the clinic visits

Time from APAC	Non PACG (n:41)	PACG (n:7)	P-value (t test)
Presentation	0.36 (±0.11)	0.4 (±0.08)	0.309
One week	0.36 (±0.11)	0.4 (±0.08)	0.309
One month	0.37 (±0.11)	0.43 (±0.1)	0.232
Six months	0.39 (±0.11)	0.59 (±0.11)	<0.001*
One year	0.41 (±0.12)	0.66 (±0.1)	<0.001*
Final appointment	0.4 (±0.11)	0.69 (±0.11)	<0.001*

*CDR* cup-disc ratio, *SD* standard deviation, *APAC* acute primary angle closure, *PACG* primary angle closure glaucoma

* Indicates high statistical significance.

Statistical analysis revealed that the length of symptoms before presentation and time taken to break the attack were linked to a higher risk of PACG development. Age and gender were not associated with disease progression (Table 4).

**Table 4:** Possible predicting factors for PACG development

	Non PACG (n:41)	PACG (n:7)	P-value
Mean age ± SD	69 ± 12	72 ± 9	0.449 (t test)
Female gender: number of patients	29 (74 %)	6 (86 %)	1.000 (Fisher’s exact test)
Time to presentation (days): median (quartiles)	2 (1 to 4)	7 (3 to 14)	<0.001 (Mann–Whitney test)*
Time to break the attack (hours): median (quartiles)	4 (2 to 6)	6 (5 to 18)	0.006 (Mann–Whitney test)*
BCVA < 6/60 at presentation: number of eyes	8 (20 %)	2 (29 %)	0.412 (Fisher’s exact test)

*PACG* primary angle closure glaucoma, *SD* standard deviation, *IOP* intraocular pressure, *VA* best-corrected visual acuity

* Indicates statistical significance.

The analysis of the dataset showed no significant difference in results when using one or both eyes of patients.

As expected, more interventions including repeated LPI, cataract extraction and trabeculectomy were required in the PACG group (Table 5). In all PACG eyes cataract extraction was combined with goniosynechialysis. Trabeculectomy with Mitomycin C was performed on one subject with uncontrolled IOP despite maximal medical treatment and on another individual who developed advanced glaucoma. One subject affected by advanced glaucoma was treated with cyclodiode laser ciliary body ablation because of systemic co-morbidities which prevented glaucoma filtration surgery.

**Table 5:** Interventions performed and number of eyes requiring topical hypotensive agents at the final visit

	Non PACG (n:41)	PACG (n:7)
LPI	41 (100 %)	7 (100 %)
Repeat LPI	7 (17 %)	3 (43 %)
ALPI	5 (12 %)	1 (14 %)
Cyclodiode	1 (2 %)	0 (0 %)
Cataract extraction +/− GSL	20 (49 %)	6 (86 %)
Trabeculectomy + MMC	1 (2 %)	1 (14 %)
Topical hypotensive agents	5 (12 %)	4 (57 %)

*PACG* primary angle closure glaucoma, *LPI* laser peripheral iridotomy, *ALPI* argon laser peripheral iridoplasty, *GSL* goniosynechialysis, *MMC* Mitomycin C

At the final visit 5/41 eyes that had PAC and 4/7 eyes affected by PACG required topical hypotensive agents (Table 5). In the PAC group only one eye required more than one medication. In the PACG group three eyes required more than one hypotensive agent to maintain a satisfactory IOP control.

## Discussion

Whilst over the past decade several studies have investigated the long-term vision and progression to PACG in South-East Asian individuals following APAC, very limited evidence exists on White Caucasian populations. In addition, most White Caucasian series used IOP control and final BCVA as main outcome measures without reporting VF data and the number of eyes which progressed to PACG.

It is controversial whether or not a single episode of APAC can lead to glaucomatous optic neuropathy (GON).

Previous publications reported that one APAC attack can result in significant retinal nerve fibre layer (RNFL) defects but not in an increased CDR [6, 7]. However, these studies had either a short follow up period of only six weeks or they excluded cases with uncontrolled IOP following the acute event. This might have led to the omission of some cases affected by GON. In our series the mean final visit was over two years and the mean IOP at the one and six-month appointment in the PACG group was significantly higher than in PAC eyes. This might have led to GON and the increased CDR at the six- and twelve-month visit in keeping with previous publications describing PACG development and mean CDR worsening weeks to months after APAC [8, 9].

There is limited evidence in the literature about the percentage of APAC eyes which develop PACG in White Caucasian populations. In our series 15 % of the eyes had PACG at the final visit.

Two previous publications on White Caucasians found that one third of patients had PACG at least six months following APAC [10, 11]. However, cases of PACG at presentation were not excluded which might account for the higher morbidity compared to our study.

In a randomised controlled trial assessing the outcomes of LPI versus surgical peripheral iridectomy (SPI) only 12.5 % of the eyes developed PACG three years after APAC [12]. Although the authors did not include subjects affected by glaucoma at presentation, they excluded all eyes with uncontrolled IOP after the initial treatment.

To our knowledge, we report the first study in White Caucasians investigating the risk factors which contributed to PACG development after APAC in eyes not affected by glaucoma at presentation. In South-East Asians, Tan et al. found that 21 % of eyes developed PACG over a similar time frame to our study [13]. Duration of symptoms before presentation and time to break the attack were associated with an increased risk of progression to PACG while mean age and initial IOP were not. Although only 15 % of eyes developed glaucoma in our series, we found the same factors to be associated with PACG development.

Delayed presentation and/or the time taken to control the acute attack were also associated with poor final visual outcome in previous studies and correlated with long-term IOP elevation and the subsequent need for further interventions after LPI [8, 14–16]. Higher morbidity in cases suffering a longer APAC attack might be due to more extensive peripheral anterior synechiae (PAS) formation and pigment release which can compromise the drainage mechanism of the trabecular meshwork.

Other studies, however, could not identify any risk factors for PACG development, a poor visual outcome or the need for more aggressive treatment [3, 10, 17, 18]. Nevertheless, the inclusion of glaucomatous eyes at presentation in these series was a confounding factor.

Finally, previous studies on White Caucasians found a reduction in BCVA in 12 % to 24 % of eyes several years after APAC [10, 14, 17–21]. Recent publications in South-East Asians reported rates between 0 % and 17 % [3, 8, 13, 22]. APAC or PACG were directly responsible for the poor BCVA in up to 60 % of these cases [3, 10, 13, 17, 18, 20, 21].

The inconsistent results are due to the definition of poor vision chosen by the authors (the BCVA cut-off ranged from ≤ 6/60 to < 3/60), the variability in the last follow up visit which was from six months to over six years, the ethnic differences of angle closure mechanism and the continuous improvement in the management of this disease. In addition, only Tan et al. excluded cases of PACG at presentation which might explain the considerably better long-term BCVA of their and our series [13].

We found only one previous study published in 1975 assessing the long-term VF outcome in White Caucasians [20]. The authors reported that a scotoma was present in nearly 40 % of the affected eyes one year after the acute event. In South-East Asians the percentage ranged from 32 % to 38 % [8, 23]. A severely constricted VF of less than 10 degrees from the point of fixation was found in 5.6 % of the APAC eyes in a large series by Aung et al. while in our cohort this was the case in 4 % of the eyes [3]. Sng et al. reported a similar mean MD compared to our data but a higher incidence of glaucomatous scotoma of approximately 32 % possibly due to the longer follow up period considered in their series [23].

## Conclusion

In conclusion, in our sample of White Caucasian patients delayed presentation to the emergency department and longer time to break the attack were linked to an increased risk of developing PACG. Therefore an improved public awareness and rapid referrals from other healthcare professionals could lead to a reduction in the incidence of PACG. In addition, APAC cases should be managed promptly according to an established protocol which should include rapid escalation to laser or surgical treatment when the attack cannot be broken with medications. Patients should be closely monitored particularly during the first six months after APAC as we demonstrated that the chance of an IOP raise is greater during this time. Tighter IOP control might prevent PACG development.

We understand that our study has limitations. It is a retrospective series with a small sample size. In addition, CDR progression analysis was based on the clinicians' judgment as the number of subjects who had objective investigations to assess the optic disc and RNFL was not sufficient for statistical testing. Nevertheless, to the best of our knowledge, this is the first study involving White Caucasian eyes without glaucoma at presentation which assessed the long-term visual disability after APAC and the risk factors leading to PACG development.
